# Through-the-Wall Microwave Imaging: Forward and Inverse Scattering Modeling

**DOI:** 10.3390/s20102865

**Published:** 2020-05-18

**Authors:** Alessandro Fedeli, Matteo Pastorino, Cristina Ponti, Andrea Randazzo, Giuseppe Schettini

**Affiliations:** 1 Department of Electrical, Electronic, Telecommunications Engineering, and Naval Architecture, University of Genoa, 16145 Genoa, Italy; alessandro.fedeli@unige.it (A.F.); matteo.pastorino@unige.it (M.P.); 2 Department of Engineering, “Roma Tre” University, via Vito Volterra 62, 00146 Rome, Italy; giuseppe.schettini@uniroma3.it; 3 National Interuniversity Consortium for Telecommunications, Roma Tre University, 00146 Rome, Italy

**Keywords:** electromagnetic scattering, buried objects, through-wall radar, microwave imaging, inverse scattering

## Abstract

The imaging of dielectric targets hidden behind a wall is addressed in this paper. An analytical solver for a fast and accurate computation of the forward scattered field by the targets is proposed, which takes into account all the interactions of the electromagnetic field with the interfaces of the wall. Furthermore, an inversion procedure able to address the full underlying non-linear inverse scattering problem is introduced. This technique exploits a regularizing scheme in Lebesgue spaces in order to reconstruct an image of the hidden targets. Preliminary numerical results are provided in order to initially assess the capabilities of the developed solvers.

## 1. Introduction

Microwave imaging of targets placed behind a wall is a topic of great interest in the remote detection of humans or objects in indoor environments, with applications in surveillance, rescue, and defense [1]. The instrument usually adopted in this class of surveys is the so-called through-wall (TW) radar, which includes a wide set of possible hardware architectures. For example, as regards the source of the interrogating field, a possible solution is to use wideband antennas radiating pulsed electromagnetic (EM) fields, or to adopt frequency-stepped sources, radiating an EM field at a limited number of frequencies.

Beyond the radar architecture, the processing of experimental data plays an important role. In most processing approaches, the goal is to localize hidden targets or produce a qualitative image of the indoor scenario [1,2,3,4,5,6], returning information about the target’s shape or the presence of interfaces. Accurate forward scattering approaches may improve the reconstruction capabilities of radar surveys. First, the synthetic data obtained by the numerical modeling are helpful in providing a deeper physical insight on the fields scattered by typical TW targets, in several practical cases. Second, the theoretical solution to the forward scattering problem is a useful tool in imaging approaches, especially when aiming for a quantitative reconstruction of the target. In this respect, forward solvers find twofold applications. On the one hand, the numerical data they provide can be used as input data to validate inversion schemes. On the other hand, forward solvers can be employed as building blocks of non-linear inversion algorithms themselves. In this perspective, the forward scattering solver should develop a full-wave solution, where all the effects of the wall interfaces on the propagation of the scattered field are suitably modeled. As for the forward solvers in TW problems, the methods proposed in the literature are essentially numerical [2,3,7,8,9,10]. Due to its high flexibility, the finite-difference time-domain (FDTD) method is mainly adopted, and, being directly developed in the time-domain, its application to TW radars with pulsed sources is straightforward [7,8,9,11]. When modeling stepped-frequency sources, frequency domain data should be computed through an inverse fast Fourier transform [2]. However, due to the large size of investigation domains in TW settings, the FDTD modeling for frequency domain analysis may be demanding in terms of execution times and memory requirements. As for the frequency-domain techniques, other methods such as the ones based on method-of-moments (MoM) approaches may be employed, which are still numerical [3]. Asymptotic techniques are also used for modeling very large regions [10]. However, when applied to imaging approaches, forward solvers are usually implemented through linearized formulations [2,3,12,13,14] or by using synthetic aperture schemes [4,5,15], thus leading to qualitative images of the target. Techniques to improve the spatial resolution [16,17] and human discrimination [18,19] have also been proposed, through frequency spectral analysis. The implementation of full-wave inverse scattering approaches for quantitative TW imaging is still an open issue, as most algorithms for solving the non-linear inverse scattering problem are usually developed for free-space applications [20,21,22,23,24,25,26,27,28,29,30] and must be properly adapted to include the presence of the wall. 

In this paper, the issues relevant to the modeling of the forward scattering problem and to the quantitative imaging are both addressed. In particular, a single-frequency non-linear inversion procedure based on a regularization scheme in Lebesgue-spaces is proposed. This kind of inversion strategy was initially developed in free-space environments [31,32,33,34,35] and subsequently extended to the case of targets buried in a homogeneous soil [36], showing good reconstruction capabilities. It has been found that the geometrical properties of Lebesgue-space norms lead to regularized solutions endowed with less oversmoothing than classical Hilbert-space regularization schemes, improving both target localization and their shaping. In this work, the method is extended to the case of targets hidden behind a dielectric wall, by modifying the underlying scattering model in order to include the proper Green’s function for layered media [7]. The validity of this inversion scheme is assessed in its forward scattering formulation, as well as in its application to the imaging procedure. For the validation of the proposed technique, an analytical solver has been employed, called the cylindrical wave approach (CWA) [37,38,39,40,41]. It provides an analytical/numerical solution to a layout with buried targets, given by circular cross-section cylinders, employing cylindrical waves as basis functions of the scattered field. Due to the presence of the interface, suitable cylindrical waves are introduced, defined through spectral integrals, to deal with reflection and transmission of the scattered field by the interfaces. A preliminary implementation of the CWA for TW scattering was proposed in [38], with an approach through multiple reflection fields to accurately describe the multipath inside the wall. In [39], the CWA has been developed through a non-iterative approach, where all the multiple interactions were included in suitable reflection and transmission coefficients. The same technique has been applied in [41], where a pulsed source field has been modeled. In this work, the results provided by the analytical solver are validated considering an integral formulation based on a Green’s function approach, and they are used as reference data to test the reconstruction capabilities of the inversion procedure. It is worth remarking that the novelty of the paper is both on inverse and forward modeling. From the point of view of the inversion procedure, an efficient technique working in the framework of Lebesgue spaces (which was developed for free-space and half-space scenarios) is here extended to through-the-wall configurations. Specifically, the presence of the wall is considered by inserting the proper Green’s function into the scattering model. In this way, the through-the-wall propagation phenomena are fully taken into account, even under near-field conditions (differently from synthetic aperture and beamforming schemes, where far-field conditions are usually assumed). Moreover, the adopted scattering model does not rely on approximations (e.g., the Born or Kirchhoff models often adopted in TW imaging). However, the presence of multiple interfaces and the availability of few limited-view measurements (i.e., only along one side of the wall) significantly increase the difficulty of the inverse problem. Consequently, the present paper is aimed at evaluating the regularization properties of the approach even in this more involved case. Moreover, an automatic criterion for selecting the optimal Lebesgue-space norm parameter based on the entropy principle is proposed for the first time. The analytical solver used in the validation of the inversion procedure is implemented for a monochromatic line-current source and dielectric targets, applying the spectral-domain analysis developed in [39] to a more realistic source. In the proposed approach, the total field is decomposed into two different sets: scattered fields by the cylinders, and non-scattered fields, i.e., the field radiated by the line source and the fields excited by its reflection and transmission at the interfaces, in the absence of the cylinders. The non-iterative approach is applied to both sets and, through suitable reflection and transmission coefficients, all the multiple interactions of the fields inside the layer are collected in two contributions: an up-ward and a down-ward propagating wave. Therefore, a theoretical solution is developed through a very compact formulation, with the total field in each medium decomposed in a limited number of terms. This approach leads to a numerical implementation which is fast and efficient.

The paper is organized as follows: in Section 2, an overview of the proposed forward and inverse scattering approaches is reported. Forward and inverse scattering results are then presented in Section 3. Conclusions follow in Section 4.

## 2. Theoretical Approach to the Through-Wall Imaging Problem

**Figure 1:**
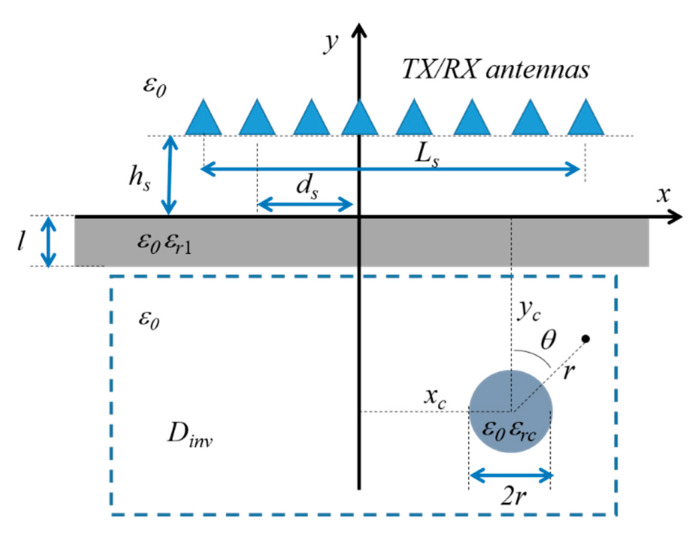
Configuration of the scattering problem, with one target placed behind a dielectric wall.

The geometry of the TW imaging problem is shown in Figure 1. A two-dimensional layout is considered, with one lossless dielectric wall between two semi-infinite regions filled with air (i.e., characterized by the vacuum dielectric permittivity, $\varepsilon_{0}$). The wall has relative permittivity $\varepsilon_{r1}$ and thickness l. The hidden investigation domain $D_{inv}$, highlighted by the dashed box in Figure 1, is located in the medium behind the wall, and contains one infinitely long cylinder with circular cross section having center in $\left(x_{c},\;y_{c}\right)$, radius a, and relative permittivity $\varepsilon_{rc}$.

A set of M transmitting/receiving antennas placed along a line of length $L_{s}$ parallel to the interface at a fixed distance $y=h_{s}$ in front of the wall is used. The transmitting antennas are modeled by monochromatic line-current sources with angular frequency ω, and it is assumed that a ${\mathrm{TM}}_{z}$-polarized incident electric field $E_{inc}=E_{inc}\left(x,y\right)\hat{z}$ is excited. The expression of the field radiated by the transmitting antennas with center in $\left(d_{s},h_{s}\right)$ is given by [42]:

(1) $$E_{inc}\left(x,y\right)=V_{0}H_{0}^{\left(2\right)}\left(\sqrt{{\left(x-d_{s}\right)}^{2}+{\left(y-h_{s}\right)}^{2}}\right)$$

where $H_{0}^{\left(2\right)}\left(\cdot \right)$ is the zero-th order second-kind Hankel function, and $V_{0}$ is the complex amplitude of the field. The $e^{j\omega t}$ term is omitted throughout the paper.

Antennas are scanned in a multi-illumination multi-view configuration, i.e., each antenna is used in turn in transmission mode to radiate the incident electric field in (1), and the total field $E_{tot}=E_{tot}\left(x,y\right)\hat{z}$ produced by the interaction of the EM wave with the investigation domain (including the wall and the target) is received by the remaining M−1 antennas. It is worth remarking that the assumed scattering model is formally exact only when dealing with cylindrical targets (i.e., ideally infinite and invariant along the z direction) under a ${\mathrm{TM}}_{z}$ illumination. In practical TW imaging applications, the inspected objects, as well as the wall, although not being infinite are usually elongated along the vertical direction (corresponding to the *z* axis in our settings). Consequently, the predicted fields are sufficiently accurate for solving the imaging problem at hand.

### 2.1. Forward-Scattering Problem Formulation

The theoretical method adopted to evaluate the scattered field in the layout of Figure 1 is the cylindrical wave approach [39,41]. The total field $E_{tot}=E_{tot}\left(x,y\right)\hat{z}$ is given by the superposition of two sets of fields. The field radiated by the transmitting antenna in (1) and the fields relevant to its reflection and transmission from the interface (in the absence of the target) belong to the first set, representing known field contributions. The second set of fields is given by the scattered field by the target in the medium behind the wall, and by the scattered-reflected and transmitted fields through the wall interfaces. In the lowest medium, the scattered electric field is found from $E_{scatt}\left(x,y\right)=E_{tot}\left(x,y\right)-E_{t2}\left(x,y\right)$, where $E_{t2}\left(x,y\right)$ is the field related to the transmission of the incident field $E_{inc}\left(x,y\right)$ in the medium behind the wall. The scattered field in the medium behind the wall is given in turn by the superposition of three contributions, $E_{s}\left(x,y\right)$, $E_{sr}\left(x,y\right),\;E_{sc}\left(x,y\right)$, i.e,

(2) $$E_{scatt}\left(x,y\right)=E_{s}\left(x,y\right)+E_{sr}\left(x,y\right)+E_{sc}\left(x,y\right)$$

where $E_{s}$ represents the field scattered by the target, $E_{sr}$ is the scattered-reflected field, describing the reflection of the field $E_{s}$ by the wall, and $E_{sc}$ is the contribution of scattered field that is transmitted inside the cylinder.

The scattered field $E_{s}$ in (2) is expressed through an expansion into a series of basis functions $CW_{m}$ [37]:

(3) $$E_{s}\left(x,y\right)=V_{0}\overset{+\infty }{\underset{m=-\infty }{\sum }}c_{m}CW_{m}\left(x,y\right)$$

where $c_{m}$ are unknown expansion coefficients and the basis functions $CW_{m}$ are cylindrical waves, proportional to *m*-th order Hankel functions:

(4) $$CW_{m}\left(x,y\right)=H_{m}^{\left(2\right)}\left(k_{0}r\right)e^{jm\theta }$$

where (r, θ) are polar coordinates centered on the cylinder.

The use of cylindrical waves as functions of expansion of the fields scattered by circular cross-section cylinders gives the analytical basis to the method. However, as the target is not in free space, but placed behind a dielectric wall, the interaction with the wall interfaces in terms of reflection and transmission must be suitably modeled. This is accomplished by expressing the cylindrical waves in (4) through an alternative definition, i.e., the plane-wave spectrum of a cylindrical wave:

(5) $$CW_{m}^{}\left(x,y\right)=\frac{1}{2\pi }\int_{-\infty }^{+\infty }F_{m}\left(y,k_{\left|\right|}\right)e^{-jk_{\left|\right|}x}dk_{\left|\right|}$$

where $F_{m}\left(y,k_{\left|\right|}\right)$ is the plane-wave spectrum:

(6) $$F_{m}\left(y,k_{\left|\right|}\right)=-\frac{2e^{-j\left|y\right|\sqrt{1-{\left(k_{\left|\right|}\right)}^{2}}}}{\sqrt{1-{\left(k_{\left|\right|}\right)}^{2}}}\{\begin{array}{c} e^{jm\cos^{-1}n_{\left|\right|}},\;\;\;\;y\geq 0\\ e^{-jm\cos^{-1}n_{\left|\right|}},\;\;\;\;y\leq 0 \end{array}$$

The expressions (5) and (6) are used to derive the scattered-reflected field $E_{sr}\left(x,y\right)$ in (2) and the scattered fields propagating inside the wall and in the first half-space [39]. In particular, in the half-space in front of the wall, where the field is probed by the receiving antennas, the scattered field is found as $E_{scatt}\left(x,y\right)=E_{tot}\left(x,y\right)-E_{inc}\left(x,y\right)-E_{r1}\left(x,y\right)$, where $E_{r1}\left(x,y\right)$ is the contribution relevant to the reflection of incident field by the interface in y=0. The scattered field $E_{scatt}\left(x,y\right)$ is defined through the following expansion [39]:

(7) $$E_{scatt}\left(x,y\right)=V_{0}\overset{+\infty }{\underset{m=-\infty }{\sum }}c_{m}TW_{m}^{0}\left(x,y;y_{c}\right)$$

where the basis functions $TW_{m}^{0}\left(x,y;y_{c}\right)$ are transmitted cylindrical waves, and they are expressed through spectral integrals:

(8) $$TW_{m}^{0}\left(x,y;y_{c}\right)=\frac{1}{2\pi }\int_{-\infty }^{+\infty }T_{10}\left(k_{\left|\right|}\right)T_{21}\left(k_{\left|\right|}\right)F_{m}\left[n_{2}\left(y_{c}-l\right),k_{\left|\right|}\right]e^{-jy\sqrt{k_{0}^{2}-{\left(n_{2}k_{\left|\right|}\right)}^{2}}}e^{-jk_{\left|\right|}\left(x-x_{c}\right)}dk_{\left|\right|}$$

In (8), $T_{10}\left(n_{\left|\right|}\right)$ and $T_{21}\left(n_{\left|\right|}\right)$ are the transmission coefficients from the wall to the upper medium and from the lowest medium to the wall, respectively. In the expression (8), all the multiple reflections excited by propagation of the scattered field $E_{s}$ in the wall are included through transmission and reflection coefficients related to the interaction of a plane wave with a dielectric slab [43]. A solution to the scattering problem is developed imposing the boundary conditions of continuity of the field components tangential to the cylinder’s interface and deriving the unknown expansion coefficients $c_{m}$ in (3) and (8) [39].

### 2.2. Inverse-Scattering Problem Formulation

In the inversion procedure, the space-dependent dielectric properties of the investigation domain $D_{inv}$ are described by the contrast function $c\left(x,y\right)=\varepsilon \left(x,y\right)/\varepsilon_{0}-1$, ε(x,y) being the dielectric permittivity in a generic point $\left(x,y\right)\in D_{inv}$, which represents the unknown to be retrieved. Such a quantity is related to the scattered field $E_{scatt}$ in the measurement points by means of the following integral relationship (data equation) [21]

(9) $$E_{scatt}\left(x,y\right)=G_{w}^{ext}\left(cE_{tot}\right)\left(x,y\right)=-k_{0}^{2}\int_{D_{inv}}c\left(x^{\prime },y^{\prime }\right)E_{tot}\left(x^{\prime },y^{\prime }\right)g_{w}\left(x,y,x^{\prime },y^{\prime }\right)dx^{\prime }dy^{\prime }$$

where $k_{0}=\omega {\left(\varepsilon_{0}\mu_{0}\right)}^{0.5}$ is the vacuum wavenumber and $G_{w}^{ext}$ is a linear integral operator whose kernel is the two-dimensional Green’s function of the considered three-layer background, $g_{w}$, which is given by [7]

(10) $$g_{w}\left(x,y,x^{\prime },y^{\prime }\right)=\frac{j}{4\pi }\int_{-\infty }^{+\infty }\frac{e^{j\zeta \left(x-x^{\prime }\right)}}{\gamma_{1}}\{\begin{array}{c} e^{-j\gamma_{0}\left|y-y^{\prime }\right|}+Re^{-j\gamma_{0}\left(y+y^{\prime }\right)},\;\;\;\;y\geq 0\\ Te^{j\gamma_{0}\left(y+l-y^{\prime }\right)},\;\;\;\;y\leq -l_{w} \end{array}d\zeta$$

where $\gamma_{0}=\sqrt{k_{0}^{2}-\zeta^{2}}$ and

(11) $$R=\rho_{w}\frac{1-e^{-2j\gamma_{1}l}}{1-\rho_{w}^{2}e^{-2j\gamma_{1}l}},\;\;T=\frac{\left(1-\rho_{w}^{2}\right)e^{-j\gamma_{1}l}}{1-\rho_{w}^{2}e^{-2j\gamma_{1}l}}$$

with $\gamma_{1}=\sqrt{k_{1}^{2}-\zeta }$, $k_{1}=k_{0}\varepsilon_{r1}^{0.5}$ being the wavenumber in the wall, and $\rho_{w}=\left(\gamma_{0}-\gamma_{1}\right)/\left(\gamma_{0}+\gamma_{1}\right)$. For the sake of simplicity, a single view case is considered in this Section. The total electric field $E_{tot}\left(x^{\prime },y^{\prime }\right)$ inside the integral in (9) depends itself on the contrast function c and can be expressed by means of a second integral equation similar to (9) (the so-called state equation), i.e., $E_{tot}\left(x,y\right)=E_{inc}\left(x,y\right)+G_{w}^{int}\left(cE_{tot}\right)\left(x,y\right)$, where $G_{w}^{int}$ is again a linear integral operator whose kernel is the Green’s function for the through-wall configuration [21]. By combining the data and state equations, the inverse scattering problem can be finally formulated as [21]

(12) $$E_{scatt}\left(x,y\right)=T\left(c\right)\left(x,y\right)=G_{w}^{ext}c\left(ℐ-G_{w}^{int}c\right)E_{inc}\left(x,y\right)$$

The non-linear problem at hand is solved in a regularized sense by using an inversion procedure developed in the framework of Lebesgue spaces $L^{p}$, i.e., function spaces endowed with the norm ${\left\|u\right\|}_{L^{p}}^{p}=\int^{\;}{\left|u\left(x,y\right)\right|}^{p}dxdy$ (u being a generic function belonging to $L^{p}$). It is worth noting that the norm exponent p represents an additional parameter that can be tuned in order to enhance the reconstruction performance. In particular, the developed procedure is based on an iterative outer–inner Newton scheme, which can be summarized by the following steps [31,33]:

1. Set the outer iteration index to n=0 and initialize the contrast function at the first outer step with $c_{0}=0$.
2. Linearize the scattering problem by computing the Fréchet derivative $T_{n}^{\prime }$ of the operator T around the current solution $c_{n}$. A linear problem $T_{n}^{\prime }\xi_{n}\left(x,y\right)=E_{scatt}\left(x,y\right)-T\left(c_{n}\right)\left(x,y\right)$ is then obtained. It is worth remarking that, similarly to the corresponding procedures in free space [31,33], the computation of the right-hand side of the linear problem and of the Fréchet derivative $T_{n}^{\prime }$ requires the solution of a set of forward problems. To this end, a forward solver based on the MoM is adopted.
3. Solve the obtained linear problem in a regularized sense by means of the Lebesgue-space procedure detailed in [31,33]. Specifically, the solution of the linear problem obtained in step 2, i.e., $\xi_{n}$, is computed by means of the following Landweber-type iterations:
  (13) $$\xi_{n,l+1}=J_{q}\left(J_{p}\left(\xi_{n,l}\right)-\beta T_{n}^{’*}J_{p}\left(T_{n}^{’}\xi_{n,l}-E_{scatt}\left(x,y\right)+T\left(c_{n}\right)\right)\right)$$
  where $\xi_{n,0}=0,$ $\beta =\|T_{n}^{\prime }{\|}_{2}^{-2}$ is a relaxation coefficient, q=p/(p−1) is the Hölder conjugate of p, and the duality map $J_{p}$ is defined as $J_{p}\left(e\right)={\left\|e\right\|}_{p}^{2-p}{\left|e\right|}^{p-1}\mathrm{sign}\left(e\right)$, with sign(e)= e/|e| (if e≠0, otherwise it is equal to zero).
4. Update the contrast function by adding the solution of the linear problem $\xi_{n}$ found at step 3 to the current value, i.e., $c_{n+1}=c_{n}+\xi_{n}$
5. Iterate from step 2 until a proper stopping criterion is satisfied.

## 3. Numerical Results

### 3.1. Validation of the Forward Methods

**Figure 2:**
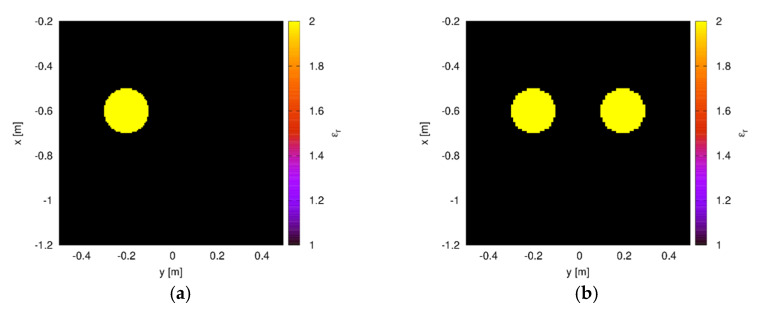
Actual configuration. (**a**) Single dielectric cylinder and (**b**) two separate dielectric cylinders.

**Figure 3:**
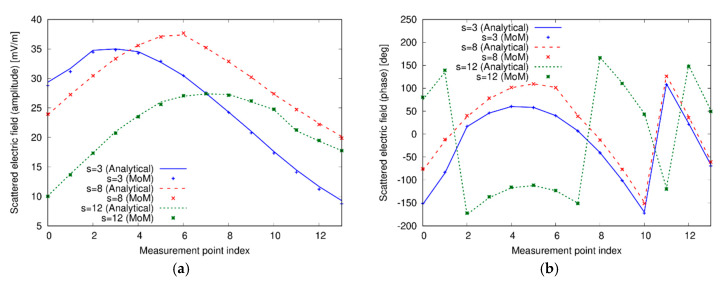
(**a**) Amplitude and (**b**) phase of the scattered fields in some of the considered view computed by the analytical forward solver based on the cylindrical wave approach (CWA) and by the integral equation formulation used in the inverse scattering procedure. Single dielectric cylinder.

A comparison between the analytical TW solver (Section 2.1) and the forward scattering model embedded inside the inversion procedure (Section 2.2) is reported here, for a cross-validation of the two forward approaches. A multistatic and multiview configuration has been simulated, with M=15 transmitting and receiving antennas aligned in front of the wall along a line of length $L_{s}=1.5\;m$, with spacing $d=L_{s}/\left(M-1\right)$, and parallel to the wall at distance $h_{s}=30\;\mathrm{cm}$. The s-th transmitting antenna (s=1,…, M) is placed along the horizontal axis in the following position:

(14) $$x_{s}^{TX}=-\frac{L_{s}}{2}+\left(s-1\right)d$$

whereas the scattered field is probed at the remaining M−1 positions along $L_{s}$. The working frequency has been fixed equal to 1 GHz. As a first case, a single dielectric cylinder with center in (−20 cm, 60 cm), radius a=10 cm, and relative permittivity $\varepsilon_{rc}=2$, placed behind a wall of relative permittivity $\varepsilon_{r1}=4$ and thickness l=20 cm, has been considered. The actual distribution of the relative dielectric permittivity in the investigation domain is shown in Figure 2a. In the MoM solver, the target has been discretized into N=900 square subdomains of side about equal to 6.7 mm. In the CWA, the order m of the cylindrical waves in Equation (7) has been truncated to $M_{t}=9$, being the total number of terms in the cylindrical expansions equal to $2M_{t}+1$. The truncation order has been determined applying the rule $M_{t}=3\sqrt{\epsilon_{r1}}a\left(2\pi \right)/\lambda$, that allows a compromise between accuracy and computational heaviness. Figure 3 shows the amplitude and phase of the fields computed by the two approaches for some of the considered views, at the M−1 measurement receiving points. Plots are evaluated for different values of the index s, which denotes the antenna used in the transmission mode, according to Equation (14). As can be seen, a very good agreement between the analytical solver used in the forward approach and the solver employed in the inversion procedure is obtained.

**Figure 4:**
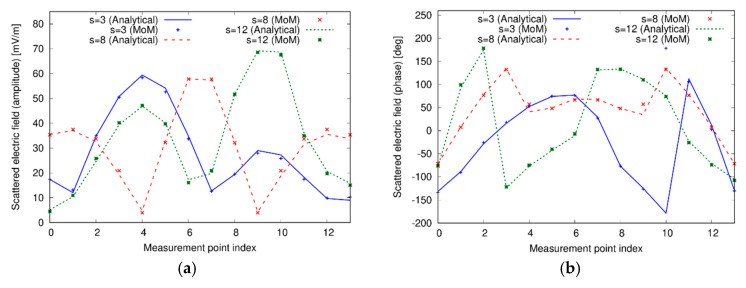
(**a**) Amplitude and (**b**) phase of the scattered fields in some of the considered view computed by the analytical forward solver based on the CWA and by the integral equation formulation used in the inverse scattering procedure. Two separate dielectric cylinders.

As a second test case, two dielectric cylinders have been considered inside the investigation domain. The first one is the same considered above, whereas the second one is a dielectric cylinder with center in (20 cm, 60 cm), radius a=10 cm, and relative permittivity $\varepsilon_{rc}=2$. The corresponding distribution of the relative dielectric permittivity in the investigation domain is shown in Figure 2b. Figure 4 reports the amplitude and phase of the field computed by using the CWA and the MoM approaches. In this more complex case, too, there is a good agreement between the two solving schemes, confirming the correctness and suitability of the analytical and numerical solvers adopted in the data generation and inversion steps.

### 3.2. Inversion Scheme

Some preliminary examples of reconstructions provided by the previously described inversion procedure are reported in this Section. In particular, the two configurations adopted for the comparison in the previous Section are considered. In order to simulate a more realistic scenario, a Gaussian noise with zero mean value and variance corresponding to a signal-to-noise ratio of 20 dB has been added to the computed scattered electric field data. The following values of the algorithm’s parameters have been used: p∈[1.1,2.5]; maximum number of outer iterations, 10; maximum number of inner iterations, 50; iterations stopped when the relative variation of the residual falls below the threshold 0.005. Such values have been empirically selected, based on the previous experience on Lebesgue-space inversion in the free-space scenario. The optimal value of the norm parameter p has been found by performing a sweep in the assumed range of values and by selecting the one providing the maximum entropy. Such a choice has been made since, for this particular application, it is expected that localized targets are usually present inside the inspected scenario, and consequently the sharpness of the image, which is related to its entropy, may represent a discriminating feature [44,45].

**Figure 5:**
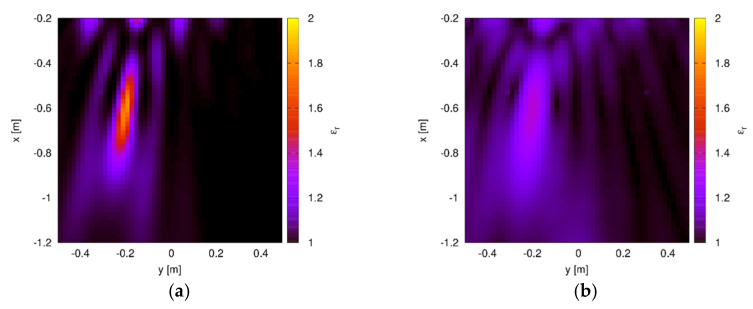
Reconstructed distribution of the relative dielectric permittivity inside the through-wall (TW) investigation domain. Single dielectric cylinder. (**a**) Optimal value of the norm parameter ($p_{opt}=1.3$) and (**b**) standard Hilbert-space approach (p=2 ).

Figure 5 shows the reconstructed distribution of the relative dielectric permittivity retrieved by the developed inverse scattering procedure. In particular, the reconstruction obtained with the optimal value of the norm parameter, i.e., $p=p_{opt}=1.3$, is reported in Figure 5a. This result evidences a correct localization of the target. Indeed, the estimated center of the cylinder is (−19.9 cm,−61.4 cm), which corresponds to an average percentage error of 1.3%. Moreover, the reconstructed value of the dielectric permittivity is close to the actual one. Specifically, the maximum value of the estimated permittivity is 1.81, which compares very well with the actual value of 2. Nevertheless, the target shape, which is a circular one, is elongated along the range direction and the cross-range size is underestimated. Such a behavior can be ascribed to the use of data collected at a single frequency, as well as to their aspect-limitedness. However, it is worth noting that even with such a small number of available data and considering just a single working frequency, the approach is able to effectively provide a quite accurate indication about the target. For comparison purposes, the reconstruction obtained by using a standard inversion procedure in Hilbert spaces (corresponding to p=2) is provided in Figure 5b. In this case, the target is still visible (the average percentage error on the center position is 2.9%), but the dielectric permittivity is significantly underestimated (the maximum value is 1.35). Moreover, stronger artifacts are present in the background.

**Figure 6:**
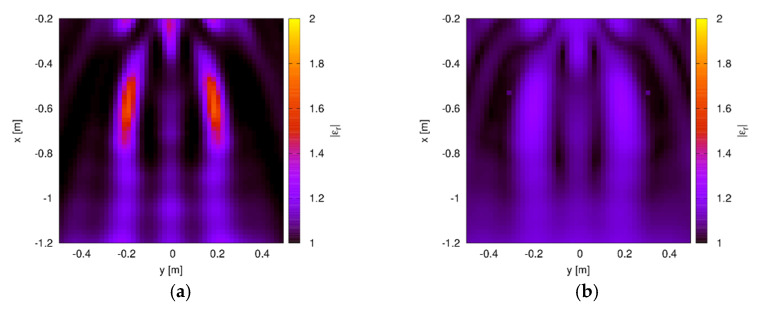
Reconstructed distribution of the relative dielectric permittivity inside the TW investigation domain. Two dielectric cylinders. (**a**) Optimal value of the norm parameter ($p_{opt}=1.3$) and (**b**) standard Hilbert-space approach (p=2 ).

**Figure 7:**
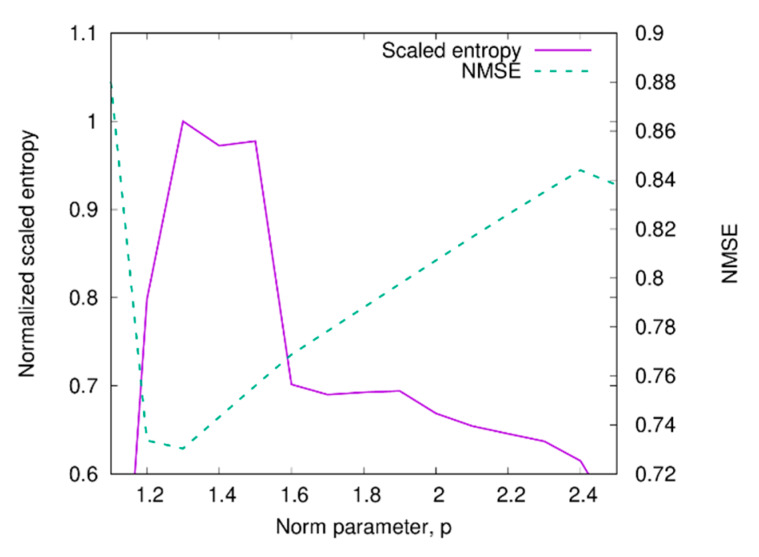
Behavior of the scaled entropy and of the reconstruction error versus the norm parameter.

As a second test case, the scattering data from the two dielectric cylinders considered in the previous Section have been used. In this case, too, the scattered field has been corrupted with a Gaussian noise with zero mean value and variance corresponding to SNR=20 dB. The parameters of the inversion procedure are the same as in the previous case. The reconstructed distribution of the relative dielectric permittivity is shown in Figure 6. In particular, Figure 6a shows the results obtained by considering the optimal value of the norm parameter, which is equal to $p_{opt}=1.3$ in this case. Even in this situation, the two targets are correctly localized, although the dielectric permittivity is slightly underestimated. Indeed, the estimated centers of the cylinders are (−19.4 cm,−57.4 cm) and (−19.0 cm,−57.0 cm), which correspond to the mean percentage errors of 3.7% and 5%, respectively, whereas the maximum values of the dielectric permittivity are both equal to 1.7. The corresponding reconstruction obtained by using the standard Hilbert-space reconstruction technique is shown in Figure 6b. Similarly to the preceding configuration, the two targets are visible (the mean percentage errors on the position are 11.2% and 7.3%), although their properties are strongly underestimated and with amplitude comparable to the background artifacts (the maximum value of the dielectric permittivity is equal to 1.26). The criterion for the selection of the optimal reconstruction has been assessed in this case by comparing the behavior of the scaled entropy with the one of the reconstruction errors (defined as $\mathrm{NMSE}=\|c-c_{act}{\|}^{2}/\|c_{act}{\|}^{2}$, $c_{act}$ being the actual distribution of the contrast function). As can be seen from Figure 7, the scaled entropy (defined as in [45]) has a maximum corresponding to the value of the norm parameter for which the lowest reconstruction error is obtained.

## 4. Conclusions

In this work, the forward scattering problem modeling and the quantitative imaging in through-wall scenarios have been addressed. For the solution of the forward EM problem, an analytical solver based on the cylindrical wave approach has been presented. The inverse problem is solved by using a non-linear regularization technique developed in the framework of Lebesgue-spaces. The suitability of the forward and inverse solvers for the problem at hand has been evaluated with preliminary numerical simulations. Future works will be mainly devoted to the extension to multi-frequency processing, in order to increase the reconstruction accuracy, and to three-dimensional configurations. Moreover, the developed forward and inverse schemes will be validated by considering experimental measurements obtained with a real hardware setup.
